# The value of program science to optimize knowledge brokering on infectious diseases for public health

**DOI:** 10.1186/s12889-018-5493-7

**Published:** 2018-05-02

**Authors:** Marissa Becker, Margaret Haworth-Brockman, Yoav Keynan

**Affiliations:** 1 Centre for Global Public Health, Rady Faculty of Health Sciences, University of Manitoba, Winnipeg, Canada; 2 National Collaborating Centre for Infectious Diseases, Rady Faculty of Health Sciences, University of Manitoba, Winnipeg, Canada; 3 Section of Infectious Diseases, Faculty of Medicine, University of Manitoba, Winnipeg, Canada; 4 Departments of Internal Medicine, Medical Microbiology and Community Health Sciences, Winnipeg, Canada

**Keywords:** Program science, Knowledge broker, Public health, Knowledge translation, Infectious diseases

## Abstract

**Background:**

Knowledge translation (KT) and related terms have variously been defined as process and as products. In this paper we contribute to debates on effective KT, specifically knowledge brokering, by describing an adaptation of Program Science that aligns with the real-world of public health activities.

**Main abstract:**

We describe an adaptation of the Program Science framework to our knowledge translation and brokering planning and projects at the National Collaborating Centre for Infectious Diseases. The systematic approach allows for layering of knowledge year to year and translating knowledge from one infectious disease content area to another. Using a recent forum on syphilis outbreaks as an example, we also demonstrate the value of using Program Science to shape the design and delivery of the knowledge brokering event.

**Conclusion:**

The use of scientific knowledge to improve public health program design, implementation and evaluation forms the basis for the program science framework. Providing the right public health information to the right audience at the right time can foster long-term outcomes of networks and new partnerships which can potentially improve delivery of public health services.

## Background

The National Collaborating Centre for Infectious Diseases (NCCID) is one of six centres established by the Public Health Agency of Canada in 2005 to strengthen public health systems by providing timely access to evidence-informed knowledge translation, products and materials that can be easily understood, applied, and implemented in public health practice and policy [1]. Knowledge translation and equivalent communications methods in the health sector have been defined variously [2], with a standard definition in Canada being, “a dynamic and iterative process that includes synthesis, dissemination, exchange and ethically-sound application of knowledge to improve the health of Canadians, provide more effective health services and products and strengthen the health care system” [3]. There have been more than 20 years of discourse on appropriate methods and processes for effective knowledge translation (KT) in health, with theory often describing process or products, and a variety of models that have not necessarily provided any greater clarity on what KT *is* [4], nor on effecting timely change in practice or policy [5]. In this paper we contribute to the debate on effective KT, specifically knowledge brokering, by describing an overall approach adopted by NCCID – a Program Science framework that is grounded in public health. We provide an example of applying that framework to a knowledge brokering event.

The breadth of the audiences and the thematic areas encompassing infectious diseases are vast, with priorities shifting as the epidemiology of infectious diseases changes globally and locally. To overcome these challenges a variety of methods are needed to facilitate mobilization of evidence and knowledge across the “know-do gap” [6] in different contexts. NCCID has evolved over time but has retained core activities including: i) identification of knowledge needs and gaps; ii) development of knowledge translation materials to address the needs of public health and iii) establishment and maintenance of networks for knowledge sharing [1]. Specifically, NCCID has positioned itself as a knowledge *broker,* creating opportunities for exchange of evidence that lead to integrating evidence in practice and policy [4, 7, 8].

Developing and sustaining inter-connected knowledge products is key for achieving longer term knowledge translation objectives and supporting the advancement of public health. Given often competing priorities, advancing knowledge translation for public health requires forward-looking and coordinated choices. To ensure that NCCID addresses priority areas, meets the needs of stakeholders and audiences, and remains nimble and responsive to emerging infectious diseases, we have adapted and applied a Program Science framework to shape our five-year plan and our annual activities, ensuring continued engagement of stakeholders, enduring relationship with our key audiences in public health and re-affirming our credibility within our thematic areas.

## Main text

Program science is the systematic application of theoretical and empirical knowledge to optimize the scale, quality and impact of public health programs [9, 10]. It has been applied as both a program and research framework with a focus on prevention of sexually transmitted infections (STI) and HIV and more recently on maternal and child health [11]. Program science was conceived as an approach to bridge the disconnect between researchers, public health programmers, and policy makers. It aims to ensure that research and science are embedded within public health programs and that public health programs drive research questions based on field-level challenges and knowledge gaps.

There are three domains to program science which mirror a program cycle: i) strategic planning; ii) program implementation; and iii) program management and evaluation (Table 1). The strategic planning domain centres on making informed decisions about priorities and resource allocation. The implementation phase then focuses on making informed decisions about ‘where’, ‘what’ and ‘how’ to deliver interventions. The third, program evaluation, requires the generation of robust evidence as part of program management. These three spheres link together to allow for an ongoing and iterative process for the re-development and re-design of programs to respond to program indicators and outcomes, and to evolving epidemics, structures and drivers of infectious diseases. Linking the three domains contributes to program improvement and responsiveness to emerging promising practices and knowledge gaps.

**Table 1:** The domains of program science for public health programs, as described by Blanchard and Aral [9]

Domains of Practice	Spheres of Evidence	Intended Outcomes
Strategic Planning	Epidemiology Transmission dynamics Policy analysis	Define prevention objectives Prioritize the right populations Match strategy to epidemic phase
Program Implementation	Efficacy and effectiveness Operations research	Select the intervention mix Implement interventions effectively
Program Management	Surveillance Monitoring and evaluation Operations research Health systems research	Achieve high coverage Maximize efficiency Alter programs when appropriate

Work led by the National Agency for the Control of AIDS (NACA) in Nigeria provides an example of a Program Science framework applied at the country level. NACA conducted epidemic appraisals – including geographic mapping and enumeration of key populations most at risk for HIV and assessing relevant behavioural patterns -- to understand local heterogeneity in the HIV epidemic. The results of these appraisals were used to develop state-specific plans, with detailed program monitoring, for HIV prevention [12, 13].

## Adapting program science for knowledge brokering

To address complex public health questions such as HIV, public health program managers and policy makers require timely evidence to inform their decision-making. NCCID adapted and applied the Program Science framework as an internal operational approach with the goal of using a systematic approach to the development and implementation of our knowledge brokering work plans.

Each year we develop activities and knowledge products for brokering with public health audiences within the Program Science framework domains: strategic planning related to the drivers of infectious diseases and identification of where burden of disease is a priority; public health responses and interventions; and monitoring and evaluation programs and policies (Table 2). This allows us to be strategic in developing a series of linked work plans so that each year builds on learnings and successes from the prior year. Specific topics are chosen based on national infectious disease priorities, such as tuberculosis and antimicrobial stewardship, while taking into consideration heterogeneity and epidemic changes at the provincial and regional level, such as with STIs and HIV. Within the framework, work plan activities within these topics might focus 1 year on strategic planning and then the following year on program implementation – or we might translate lessons from strategic planning on tuberculosis 1 year to strategic planning on HIV the following year. The ability to address topics across the domains enriches the engagement of partners and networks working towards a meaningful outcome of better public health programs. The aim of this framework adaptation is that within NCCID we can ensure that we provide knowledge and evidence that can be used in each of the Program Science domains and also be applied to other public health topics. This situates the knowledge to allow public health mangers and decision-makers to adapt comprehensive responses for complex infectious diseases in local contexts.

**Table 2:** Examples of 1 year’s topics of knowledge brokering and translation for NCCID, applied to an adaptation of the program science framework

Domains of Practice	NCCID Topics of Evidence & Knowledge	Intended Outcomes for Public Health
Drivers & Burdens of Infectious Diseases (Strategic Planning)	Drivers & burden of TB in diverse populations Drivers & burden of poor oral health in refugee populations Drivers & burden of STIs in Indigenous youth	Choose: Best strategy Right populations Right time
Public Health Responses & Interventions (Program Implementation)	Novel approaches to syphilis outbreaks in two geographies in Canada Appropriate responses for TB in the north Regional programs for Syrian refugees	Do: The right things The right way
Monitoring and Evaluation (Program Management)	Big data for outbreak management HIV Cascade treatment indicators Directory of AMS programs in Canada	Ensure: Appropriate scale Efficiency Change, when needed

In the course of adapting Program Science to our work planning, it became evident that knowledge brokering activities do not or should not always be located in one domain over another, but the framework nevertheless serves as a guide. In the following section, we illustrate this application of program science *within* a topic area using an example from work on syphilis.

Syphilis has re-emerged from a state of very low incidence in North America to 23,872 syphilis cases reported in 2015 (compared to 6,103 in 2001) in the United States [14]. Men who have sex with men (MSM) continue to account for the majority of primary and secondary syphilis cases in 2015; 14,229 (59.6%) cases were among MSM. Syphilis rates in Canada showed a dramatic increase, rising by 101.0% between 2003 and 2012, from 2.9 to 5.8 per 100,000 [15]. Although pan-Canadian data are lagging due to jurisdictional health care responsibilities, the trends appear to follow similar patterns. Over the past few years, some changes have been observed with the migration of the epidemic outside urban centres into rural and remote areas as well as shifts into heterosexual populations [14, 16, 17]. The need for public health to address these changing demographics requires translating knowledge from the urban epidemic while developing specific strategies that take into account rural, remote, northern and cultural contexts.

Responding to requests from networks of practitioners and programmers across Canada, NCCID planned an event in the fall of 2016 to provide a broader perspective of the changing epidemiology, bringing together both urban and northern and rural public health personnel [18]. In consultation with more than 35 stakeholders across the country, who frequently named colleagues who could contribute to the event, NCCID structured the meeting for urban and northern public health personnel to meet and exchange on syphilis strategies. In all, 42 participants were invited; practitioners, program coordinators, epidemiologists, researchers, policy makers, front-line workers attended from across the Canadian territories and provinces, representing diversity across geographical locations, areas of expertise, and levels of public health authority. Based on the consultations on what would be meaningful for participants, NCCID developed the agenda to foster a flow of information that started with sharing evidence and data on the shifts in the epidemic burden and drivers of disease that guided their context-specific *strategic planning* (domain one). Participants then presented cases of their *program implementation* responses (domain two) – promising practices as well as efforts that were not deemed successful. *Program monitoring and evaluation* (domain three) were not emphasized in the course of the day-and-a-half meeting, although new results from mathematical modelling of syphilis in Manitoba suggests the value of targeting urban-dwelling individuals at risk for syphilis reinfections and can inform near-future *strategic planning* and subsequent *program interventions*, demonstrating the iterative nature of program science.

NCCID’s report and analysis of the meeting (unpublished) is a summary of interventions that have been tried including those with limited effect and those that show some promise, with an analysis of contextual barriers and enablers related to these interventions. Commonalities for the northern and urban syphilis epidemics were identified, including the need to strengthen sexual health promotion and inclusive access to health services and de-emphasize “risky” sexual activity. Different interventions were also discussed, as in the priority of addressing sexual and gender-based violence in arctic and rural communities, in comparison to discussions of successful use of social media apps for urban male communities.

NCCID staff did not use the syphilis event to explicitly engage participants in an exploration of Program Science. However, we found that organizing our thinking within the framework as we prepared for the event, and in communicating the results after the event, clarified where there are knowledge gaps and opportunities for further knowledge brokering in this topic area (such as in evidence on public health monitoring and evaluations of innovative responses). NCCID’s role in creating a space for conversation between urban and northern public health players also sparked a new network of medical officers of health in rural, remote and northern Canada which will generate new occasions for translating lessons to other priority areas in communicable diseases. In our assessment, these outcomes signify real-world and pragmatic examples of closing the “know-do” gap, while at the same time fitting knowledge brokering with public health information and program cycles; outcomes which have been seen by Urquart et al. [19] but which have not typically been made explicit in any reviews of knowledge translation research to date [7]. This example also illustrates impact that extends beyond the specific activity, including future activities and networks that were fostered.

## Conclusion

The use of scientific knowledge to improve public health program design, implementation and evaluation forms the basis for the program science framework. Providing the right public health information to the right audience at the right time has the potential to improve delivery of public health services.

Knowledge translation and brokering of evidence to support decision-making in public health requires assessment of the knowledge itself, understanding of the audiences and continued engagement. Program Science provides a useful framework to guide knowledge translation work for infectious diseases and public health and allows for the iterative process that is critical for success and responsiveness of public health programs. In this paper, we describe the adaptation and application of the Program Science framework to NCCID’s work in knowledge brokering. This systematic approach allows for layering of knowledge year to year and translating knowledge from one infectious disease content area to another. The progress of projects within the thematic areas, covering the domains of program science allows for incorporation of guidance from the audience, adaptation to changing needs, and the inclusion of evolving best practices in the choice of tools and knowledge products that are provided by the knowledge broker. These allow for continued relevance and ongoing partnerships to feed into a dynamic work plan. We will monitor whether the Program Science framework facilitates approaches to the three domains that transcend different topics.

## Abbreviations

HIV: Human immunodeficiency virus
KT: Knowledge translation
MSM: Men who have sex with men
NACA: National Agency for the Control of AIDS (Nigeria)
NCCID: National Collaborating Centre for Infectious Diseases
STI: Sexually transmitted infection
